# Patient perspectives of bedside teaching in an obstetrics, Gynaecology and neonatology hospital

**DOI:** 10.1186/s12909-020-02016-5

**Published:** 2020-04-15

**Authors:** Michelle Carty, Nicola O’Riordan, Mary Ivers, Mary F. Higgins

**Affiliations:** 1 UCD School of Psychology, University College Dublin, Donnybrook, Dublin 4, Ireland; 2 Obstetrics and Gynaecology, National Maternity Hospital, Dublin 2, Ireland; 3 UCD Perinatal Research Centre, UCD School of Medicine, Obstetrics and Gynaecology, National Maternity Hospital, Holles Street, Dublin 2, Ireland

**Keywords:** Bedside teaching - medical education, Medical student

## Abstract

**Background:**

Osler taught doctors to “have no teaching without a patient for a text, and the best teaching is that taught by the patient himself”. Bedside teaching (BST) facilitates clinical practice of skills, teaches empathy, instils confidence and builds on patient-doctor relationships. However, its use has declined dramatically due to concerns regarding privacy and autonomy. Most of the research in this area concentrates on medical student or academic opinion of BST using survey based methods. This qualitative study aimed to explore the patient’s experiences and opinions of BST.

**Methods:**

With ethical approval a qualitative study was conducted using semi-structured interviews which were examined using Thematic Analysis. Patients who had participated in a BST tutorial were invited to participate and gave written consent after discussion with a study researcher.

**Results:**

Twenty-two patients were interviewed (obstetrics ante-natal [*n* = 10], obstetrics post-natal [*n* = 5] and gynaecology [*n* = 7]) ranging from ages 24-80 yrs. Four major themes were identified, with 11 sub-themes. The major themes included (i) Professional Mannerisms (ii) Privacy and Personal Wellbeing (iii) Quality of Patient Experience of BST and (iv) Clinical Experience and Learning Importance. The reaction of patients toward teaching at the bedside was altruistic and positive, with importance placed on learning.

**Conclusion:**

This research supports the concept of patient focused learning, and can reassure faculty that patients largely support its continuation as an integral component in education. Future research aims to extend this assessment to other patient groups with the aim of learning from and improving their experience.

## Background

Osler taught to “*have no teaching without a patient for a text, and the best teaching is that taught by the patient himself*” [1]. Within this framework, bedside teaching (BST) has numerous advantages for medical education, including facilitating clinical practice of skills, teaching empathy, instilling confidence, and building on patient-doctor relationships [2–5]. BST provides higher order learning where students are exposed to a holistic approach to patient care – history taking, examination skills, professional attitude and how to come to a final differential diagnosis – as well as modelling appropriate clinical care and respectful approach to patients and their families [6]. Students report it to be a useful learning activity [7], with many reporting that it was the most effective way of learning clinical skills [8]. Despite these advantages, the use of BST has decreased from 75% of clinical teaching in the 1950s to 17% in 2009 [9]. Concern has been expressed about this decline [7]**.** Postulated causes include concerns regarding the patient’s reactions [10], who holds the “power” during BST (the patient, the students or the teacher) [11] ^R^ and fears of causing discomfort to patient [12]. In addition, the changing nature of hospital care, shortened admittance times, time constraints of all participants including patients, students and teachers, teacher and student knowledge and skills, as well as increased workload of clinical staff have been suggested as factors influencing the reduction in BST [6].

Previous publications on BST have focused on clinicians or students viewpoints of BST [7, 10, 13–15]. Those that reviewed participant’s experiences reported that patients felt BST was enjoyable, increased favourable perception of physicians and trainees, and helped understanding [5, 12, 13, 15–17]. One study reported the highest rate of refusal in Obstetrics and Gynecology^12.^ – albeit in a very specific cultural context (Kuwait) - but it is also possible that this refusal rate may be due to the sensitive nature of Obstetrics and Gynaecology, or to women’s specific attitudes to BST in general (in all other specialities – other than urology – genders were mixed). Without understanding the context in which patients declined BST it is difficult to understand why they declined. These studies used a mixture of quantitative, qualitative, and mixed methods approaches, though the majority used questionnaires, potentially restricting the interpretation of context and limiting the findings to what the researchers a priori deem to be important.

In addition, many of those studies that have used qualitative research methods have focused on BST as part of clinical rounds [14–16, 18, 19] rather than as a separate planned teaching tutorial with the main focus on medical education of medical students. The patient experience of BST is therefore potentially tied into their clinical care and not considered as an independent educational experience.

This study aimed to explore patients experiences of BST for medical students using a qualitative research approach. Our hypothesis was that qualitative research would provide a richer approach to more fully explore themes identified by the patients as important, including potentially both the patients experiences of BST and their personal opinions.

## Methods

This study was performed in the National Maternity Hospital, Dublin, a tertiary level unit with more than 9000 births per year. The hospital provides undergraduate and postgraduate teaching to the full multidisciplinary maternity team.

Within this hospital two types of bedside rounds occur. The first is a midwife lead, patient centred, clinical bedside round occurring every morning and based on the principles of family centred care [14, 17]. Led by a clinical midwife manager, the ward round team consists of a consultant, senior specialist registrar (year 8–9 post-graduation), junior registrar (year 3–4 post-graduation) and Senior House Officer (year 2–3 post-graduate). Bed side teaching of doctors in training does occur on the clinical bedside round but the focus is mostly on provision of clinical care, communication with the patient and her family, and development of plans for care. Medical students rarely participate on the clinical rounds. The second bedside rounds are focused on medical education and are teaching rounds for medical or midwifery students. For medical student, these bedside rounds (tutorials) are a timetabled component of teaching, with two to three BST per week during term time (26 weeks per year). Each BST would involve attending two to three patients. (In context, the median number of patients attending for inpatient care per week would be 100 or more). It is these educational bedside teaching rounds that are the focus of this study.

Therefore, for the purposes of this study BST was defined as follows: “*A bedside tutorial when students and a supervising qualified physician collect around a patient’s bedside to discuss their medical history, symptoms and possible diagnosis*^*5*^*in the patients presence and with their direct feedback*”.

In order to prepare for BST and other clinical teaching, students undergo orientation prior to entry into Clinical Sites that includes themes of Professionalism, Etiquette while on clinical placement, Clinical Learning, and Guiding Principles of Patient Contact [20]. “Three C’s” of patient contact are taught: Choice, Consent and Confidentiality. Students are not formally provided with a script to use when obtaining consent from patients to participate in BST, but are reminded at orientation into the module that these principles should be used at all times.

Students attend this module [Obstetrics, Gynaecology and Neonatology] during their second or third year of clinical hospital placements. At this time they have experience in consenting patients to participate in BST and are familiar with the process, including respectful etiquette if patients decline. BST tutorials are led by five faculty members, ranging in BST experience from one to thirty years (median 15 years). All faculty members receive formal training in Medical Education, including teaching in a tutorial setting.

With the principles of choice, confidentiality and consent in mind, students check with ward staff regarding which patients are suitable to approach to request an interview for BST. The teaching BST occurs sometime after the clinical ward round has completed so that ward staff are familiar with the clinical situation of all patients, and so that patients are not overwhelmed. Once approved, one or two students approach the patient to ask if they would consent to taking part in the tutorial. With full consent the students obtain a medical history and perform an appropriate abdominal examination. Students return later with their supervisor and teaching group (*n* = 5–10). Students approach the patient individually to confirm that she is still comfortable to participate. If she agrees, then the students then present her history at the bedside to other students and the supervisor and demonstrate the abdominal examination.

The rate of patients declining to participate in tutorials ranges between 20 and 50% using the strategy described above. During the time of this study, educational BST for medical students continued as normal. This study added an additional, optional, step for the patient that involved an interview post participation in BST.

Before approaching patients, the researcher made contact with the tutor to confirm the BST had occurred and then contacted the clinical midwife manager or midwives on the ward. This was to protect vulnerable patients, who may have participated in BST but whose clinical condition may have changed. Exclusion criteria included those under the age of 18, unable to give consent or serious medical condition requiring immediate medical attention.

Patients were approached and, if tentatively interested, given at least 15 min to read an information sheet without the researcher present. Following informed written consent, semi-structured interviews were conducted on a one-on-one basis where the patient could talk freely about BST in response to questions in the interview guide. The interview guide is included as supplementary material; as interviews progressed and new themes developed this guide was modified to allow exploration of new themes.

Interviews were recorded using a Dictaphone, transcribed using Express Scribe Transcription and analysed using both NVivo 11 and Microsoft Excel 2016. These were used throughout the interview process. A demographic questionnaire was included at the beginning of the interview guide, including age, indication for admission, gestational age and highest educational achievement. No specific patient identifying information (name, date of birth, hospital number) were recorded so that transcripts were anonymised. Audio files were securely stored after transcription to allow review, and will be destroyed in the usual manner. Interviews took approximately 10–20 min.

The qualitative research method was Thematic Analysis [21]. This was picked as it is a “*flexible and useful research tool*” which can “*potentially provide a rich and detailed, yet complex, account of data*” [21]

The process was as follows: As the interviews progressed and there was increasing saturation of data, the interviewers took care to carefully compare findings by checking with new participants and triangulation of data. Audiotapes were transcribed verbatim by the researchers and the transcripts were entered into NVivo and coded. Transcripts were read and reread as codes were assigned by two coders (MS, MI). The data was first divided into the eight sections outlining the semi-structured interview guide. This helped with the initial assessment of the data using a deductive approach, as the pre-existing structure of the interview guide provided base concepts, ideas and topics that acted as scaffolding for analysis. Data was then coded following the individual guiding question of the interview guide, narrowing, and defining the codes that presented in the data initially. At the end of stage two, codes were identified. Theme development occurred during stage three: codes were examined inductively, looking at what the participants were saying and whether some topics were continuous throughout the interview. The codes were then observed and grouped several times while the themes were identified by the researchers. Themes were developed from data that was prevalent across interview, important and substantial. Saturation of themes was defined when refinements did not add any new substantial themes.

Trustworthiness was enhanced by two methods: method checking and triangulation [22]. A variation of member checking was used, where instead of reviewing themes with the original participants (not possible due to anonymity and high patient turnover) themes were reviewed with a different group of patients to see if they saw them as authentic. Triangulation was via researchers where two researchers initially analysed data (MS, MI) and two reviewed identified themes (NOR, MH).

The research team had previously reflected on possible personal sources of bias: as MH and NOR had participated in BST and worked in clinical academia this was identified as a possible bias in the research and as such both identified this bias and worked with other members (MC, MI) who would not have this bias. This study is reported following the SRQR standards [23].

Research interviews occurred during the placement periods of medical students (June – November 2016) shortly after the BST. Ethics approval was obtained from National Maternity Hospital and University College Dublin Ethics Committees.

## Results

This study consisted of 22 patients that attended the hospital. The division between the groups was obstetrics ante-natal (*n* = 10), obstetrics post-natal (*n* = 5) and gynecology (*n* = 7). Participants had all completed at least secondary education. Further demographics are shown in Table 1. The median time that interview took to complete was 12.09 min (range six-33 min).

**Table 1:** Demographics of participants in semi-structured interviews

Participants			***n*** = 23
**Age**		Median 30 years (20–80 years)	
**Classification**	**Obstetrics**	Antenatal	10 (45%)
		Postnatal	4 (18%)
	**Gynecology**		8 (36%)
**Educational Level Achieved**		Primary	2 (9%)
		Secondary	4 (18%)
		Tertiary	16 (73%)

Data was coded as described in the methods section. Initially (at the end of stage two), 94 codes were identified. Thematic Analysis identified four major themes and 11 sub-themes from the 94 codes of the study.

Four major themes evolved: Professional Mannerisms, Privacy and Personal Wellbeing, Quality of Patient Experience of BST and Clinical Experience and Learning Importance.

### Professional mannerisms (Table 2)

The way in which the students and doctors conducted themselves with the women, such as acting respectfully and meeting a standard of behaviour expected of medical staff.

**Table 2:** Professional Mannerisms theme and sub-themes identified

Professional Mannerisms	Positive Mannerisms experienced by patients	Open to physical examinations by students provided that they are appropriate
		Participants were enthusiastic, interactive and confident
		Balance found between professionalism and learning
		Students educationally focused
		Not intimidating
		Students were medically serious
		Students and doctors were fine, lovely
		Students and doctors were respectful, supportive and maintained boundaries
	Negative Mannerisms experienced by patients	Nervous about students performing physical exam
		Partner felt students felt awkward and nervous
		Student mannerism matters
	Observed Differences	Students seen as a member of staff
		Expectations of reassurance
		Student mannerisms refreshing

There were three subthemes:

*Positive Mannerisms Experienced by Patients.*

Positive responses to students and doctors were common. Patients consistently used terms such as: *“lovely”, “respectful”, “supportive”, “focused”.* They felt the students balanced their requirements for learning with being medically professional - *“the boundaries were maintained, it was very ethical and very respectful, really, which was the most important thing, especially when you’ve gone through what you’ve gone through, as well, as a woman”* (# 23).

*Negative Mannerisms Experienced by Patients.*

Some patients felt that their autonomy may be compromised if the wrong mannerism was portrayed. The need for autonomy was echoed by many: *“The approach, so if the student had of come over and in a certain way or tone or mannerism, (..should be no)..”you are going to“ or “this is expected of you“, It was very gentle, it was “look it will all be up to you“ … complete respect.* (# 25).”

*Observed Differences.*

One patient commented how the mannerisms of younger doctors facilitating the BST may be different to older doctors, stating: *“I do love about dealing with younger doctors, with younger doctors they’re just so different ... They just, when they come in, they’re just like wanting to know it all, that kind of thing, like before [with older doctors] they weren’t as open or they weren’t as nice. They get irritable. You’d be very on edge but not now with the younger people, they’re great.* (# 20)”.

### Personal wellbeing and privacy (Table 3)

There were two inter-related sub-themes: privacy and personal wellbeing.

**Table 3:** Personal Wellbeing and Privacy: Themes and subthemes identified

Privacy and Personal Wellbeing	Privacy	If privacy was not provided the patient would withdraw
		Patient would prefer privacy in obstetrics
		Patient not open to physical exam by students
		Patient finds it harder to engage when procedures being done
	Personal Wellbeing	Not in any pain
		Patient would not participate if risk of long term damage
		Patient would not take part if sick, stressed, things going badly
		Patient preferred qualified doctors over students
		Nothing would affect the patients decision to participate
		Patient unsure regarding child participation

#### Personal wellbeing

Patients prioritised their own personal wellbeing over their altruistic inclination to allow students to learn. The primary reason they gave for declining to participate was their own health, with one patient stating, “*I suppose it would only be personal circumstances, if I was stressed or worried about the situation maybe, I wouldn’t want a bunch of people there, when I’m stressed, that would be a personal thing*” (# 02).

#### Privacy

Patients spoke about their need for privacy and went on to explain this in the context of Obstetrics or Gynaecology (for themselves) or Neonatology (for their baby). One patient illustrated this as follows: *“I think it’s hard to say, but I think in obstetrics I probably would want the privacy and having had a new baby, I’d be very protective of who came near them”* (#9).

### Quality of patient experience of BST

Theme Three refers to how the patients felt and thought about the experience that they had received during BST (Table 4).

**Table 4:** Quality of Patient Experiences of Bedside Teaching

Quality of Patient Experience of Bed Side Teaching	Positives of Experience	Patient felt the focus was sufficient
		Patient felt she knew to say if she was or wasn’t happy about taking part
		Patient felt student presence was controlled
		Privacy and confidentiality were respected
		Expectations could not have been better met
		Passed the time
		Helpful and interesting
		Environment was private and controlled
		Expectations met
		Patient accepted students taking her history
		Patient felt sufficiently consented
		Patient felt included and involved in teaching
		The tutorial did not upset the patient
		Patient felt that they went into more detail
		Patient felt that the tutorial moved naturally
	Improvements for future BST	Patient would like a summary
		Patient felt history taking was repetitive
		Patient felt like the subject rather than included
		Patient would like classes to be held somewhere more private
		Lots of students asking questions
		Students had difficulty understanding
		To make sure patients are ok with it
		Patients felt like the students were trying to get a lot of information in a short time
		Patient recommended advising use of tutorial to patients
		Patient would prefer more one to one or smaller groups
		Patient was unsure what would happen to the
		information collected
		Patient felt she could not ask questions until the end

#### Improvements in patient experience

One point of suggestion to improve patient experience was that some patients felt overwhelmed by the volume of tutorials, students, and questions, finding that “*there is a lot of it [BST], with students coming in and asking questions*” (#12) and that at times it can be “*a little bit, kind of repetitive*” (# 14).

#### Positives of patient experience

Patients felt they had a choice in whether they participated. They felt listened to, respected and they their opinions were asked. One patient enjoyed that she was included in the learning saying: *“at the end, you know, the Professor kind of felt around the [pregnancy] bump and was showing them the three points to check and where the head is and showing them that, and that was interesting, pretty cool* (#1)”.

#### Expectations of patient experience

In general, patients expected the students to be engaged and contributing to the tutorials. They expected the supervision of a senior member, with the students not involved in major decisions or procedures.

#### Clinical experience and learning importance

This had three sub-themes (Table 5).

**Table 5:** Clinical Experience and Learning Themes and Subthemes identified

Clinical Experience and Learning	Learning structure	Group size does not affect patient
		Students shadow their own profession
		Group tutorials would be different (to one on one) and more varied
		Students would not learn as much from group tutorials
	Learning Experience for patient	Patient new to BST and unsure of what to ask or improve
		Patient felt she learned more about her own condition
		Patient did not learn anything
	Clinical Learning	Agreement with bedside teaching
		Best person to learn from is the patient
		Clinical experience is important
		Students have to learn
		Why be difficult and not let them
		Patient indifferent to group size, type, practice type
		Patient open to child participation within reason
		The patient profile (not the patient) teaches the student
		Many jobs involve learning on the job
		Patient felt history is important to case building
		Patient has information that is useful to the students
		Be more concerned if examination not done

#### Clinical learning

The importance patients placed on students’ learning clinical skills in a hospital setting was very clear. The importance of hands on experience in medical care was considered paramount: “*if you had somebody who had learned it all and [had] a 1:1 degree and came in and was suddenly faced with a patient and an emergency and had never seen anybody deal with that before in practice, that just doesn’t make sense*” (#15). Patients believed that they had information that could help students. They appreciated that the students had to learn, just as they themselves had learnt in their own jobs.

#### Learning experience

Patients were open to both male and female students. Most were also open to allowing a child to participate in BST, whether their own or figuratively, with one participant even stating that they like to *“have that physical contact whether it be examining mum or holding the baby or checking the baby you know* (#10)*”*.

There were some differences on whether patients gained knowledge themselves from participation in BST. Those who felt they did not learn were either those who had received all their information beforehand from their medical team or those who found the medical language used inaccessible. In the group that learned from BST, this most commonly occurred when BST reviewed complications or side effects.

One of the main barriers to learning was that most patients had never previously participated in BST and were unsure how they could use the learning session for their benefit or how they could get involved in the session.

#### Learning structure

There was some disparity in terms of the practicality of large group sessions. Some believed it offered opportunities for different questions - *“A group of patients and a group of students; or having a group of students instead of one or two. I think it’s different, a chance for more questions* (#7)” - others believed the hands-on experience wasn’t as valuable in larger groups as students were demoted to observational roles - *“I don’t think they would get as much out of it either, because in a group you can’t have everyone getting hands on* (#13)”.

Patients were open to allowing all student types (i.e. medical, midwifery, nursing, allied health) to be taught at the bedside though one point that patients raised was: *“you know [you have] a midwife who is there to deliver a baby, and the doctor who’s there to address complications if they arise so maybe, so maybe, it’s better for the trainee doctors to shadow doctors as oppose to midwives.* (#2)”.

Member checking with a different group of patients (*n* = 10, by MH) revealed that patients agreed that these themes were authentic to their experience of BST and trustworthy.

## Discussion

This study has shown that, within a structured process to identify and consent patients for Bedside Teaching, that they are supportive of participating as long as certain criteria are met. Their consent to participate is crucial, with consent being denied if they felt unwell, or if the students were not respectful in their approach. Confidentiality was crucial in the handling of their information. With these criteria met, women were hugely supportive of BST, identifying that they provided unique knowledge of the lived experiences of pregnancy, labour, birth or medical conditions or issues that the students could learn from. Women recognised that students should learn from people if they are going to work with people. In addition, some women found the tutorials useful in consolidating information about their own conditions or reason for inpatient care, though most reported that they had received this information already from the multidisciplinary clinical round.

Most of the studies in this area previously have used un-validated questionnaires to study patients perceptions of BST [5, 12, 13]. Qualitative research provides a possibility to make new meanings and to allow the exploration and development of themes that the researchers had not considered prior to the study [21]. The goal is therefore to “*help us understand social phenomena in natural (rather than experimental) settings, giving due emphasis to the experiences, and views of all the participants*” - it is seeking to answer “**why?**” rather than “**how many?**” [24]. Why would someone admitted into a hospital take part voluntarily in medical education of students? Using an inductive approach – moving from the data to a hypothesis – means that we can explore what people really mean and how they really behave and why. Doctors and nurses may say that by the very nature of an admission to a teaching hospital, patients are accepting of their participation in research - this may be considered arrogant and presumptive. In fact, using the iterative approach of qualitative research has shown here that patients themselves have identified that they provide an unique experience and knowledge that students can learn from, as long as their personal circumstances are respected. Patients said they would be concerned if students had no clinical experience. Patient wished to see the students learn, which may be reassuring for students who may be concerned about making mistakes. Findings that were not expected included the significance patients placed on the mannerism of students and the importance patients place on the quality of the education that the patient also experienced.

Other studies that used qualitative research methods to study patients opinions of BST have focused on BST as part of clinical care; that is, where teaching is provided as part of clinical bedside rounds under the supervision of a physician providing care to the patient. Teaching may be provided at several levels as participants will include doctors in training (residents) as well as medical students [6]. In this context patients opinions are tied into their opinions of the medical care provided to them, whether in the context of family centred rounds [18] or traditional bedside teaching rounds [16, 19]. In one study, investigating the physiological and psychological responses of patients with cardiac disease participating in bedside rounds, the psychological response was measured by the administration of an anxiety scale that showed a low level of subjective anxiety (mean score of 30 compared to a mean of 42 in general medical inpatients). A “subjective assessment” revealed that patients supported bedside presentations, but the mechanism of this assessment is not clear [16]. In contrast, the context of BST in this study was purely for the purposes of education of medical students and the mechanism for assessment is clearly described. With no additional benefit apart from altruism, patients volunteered to participate once they were well enough to do so and were treated respectfully, because they realised the value that their input could bring to the education of medical students.

A unique feature of this study is that the themes identified represents the views, opinions and experiences of women – an area often under-represented in medical research and education [25, 26]. Other studies investigating patients experiences in BST either have not reported gender [15, 18, 19] or under-represented women [5, 16] though some have shown equal representation [12, 14, 27] or over-represented [13]. What was interesting about the results from our study was the generic nature of the findings and how they could represent any speciality – we had hypothesised that women would focus on the intimate nature of Obstetrics and Gynaecology, but this did not emerge as a theme from the research.

One area that we did not focus on within the interview guide was patients attitudes towards the facilitators, or preceptors, of BST. Within qualitative research themes should emerge independent of the interview guide [21] and the only reference that was obvious was that of the difference between facilitators with regard to their age.

The responses of participants in this study illustrates how patients believed they are the best person a student can learn from. The Medical Education theory of “*Patient as Teacher*” redefines education so that patients, service users and clients, rather than clinicians, are the teachers [28–30] . “*Patients as Teachers*” have been formally graded into four different levels, from informal (passive educational role, or working with a clinician as facilitator) to formal (acting as an expert and trained teacher with a more active role in education, curriculum planning or assessment) [30] with most of the research in this area focused on the formal levels [31–35]. This study would suggest that even “traditional” patients, in an informal role, feel they are the best people to learn from.

One of the strengths of the study is that it used open-ended questions in a semi-structured interview style to allow for in-depth responses. This qualitative study ran in a single university teaching unit and as such is limited to the culture of this unit; both study type and location may affect generalisability. This can be seen as either a strength or limitation of qualitative research. If the paradigm used is that there is no one truth but multiple versions, all context specific [36], then the exploration of BST in new environments and contexts provides further information to guide educators on how to provide BST in different situations.

There are limitations of this study. The majority of participants had tertiary education and may not have accurately reflect participants with secondary or primary level education. Furthermore, the study could not assess those who did not take part in BST initially, or declined to participate in the research study after participation. Recruitment of patients to the qualitative interview after their participation in BST sessions may have introduced potential bias. It is possible that only patients with positive experiences would agree to participate in BST and that midwifery staff would steer the investigators away from patients who they knew had suboptimal experiences. We are aware of at least one patient who had a suboptimal experience who consented to participate in order that she could provide feedback on her experience in order to improve the experience for future participants, but it is possible that this may have been a factor (in reverse) for others – for example, four postnatal women declined to participate.

One of the main concerns was social desirability bias – that participants would answer questions in a manner to be viewed favourably by the researcher [37]. However, most participants had been a student in some form of placement and could empathise with the medical students. We hope that this, with the lack of monetary reward, reduced the impact of social desirability.

A secondary study based on this study is being conducted, with the aim of creating a questionnaire which can be validated and then used to assess BST in other practices and to include those who may decline BST, so that BST may be analysed in a valid way in multiple contexts. This questionnaire is being developed following the steps described in a AMEE (Association for Medical Education in Europe) guideline [37]. These steps include a thorough literature review, conducting interviews (including these interviews), synthesising literature and interviews, developing items, conducting expert validation, cognitive interviews and pilot testing. Development of this validated questionnaire can then expand this research outside of the group of those who have participated in BST and consented to be interviewed after the BST. This may then also provide insight into the percentage of patients who may decline to participate and their opinions of BST, including reasons why they may decline.

## Conclusions

There was a general sense of fulfilment from patients, with BST being approved and accepted as an important learning tool. Patients were satisfied that their opinions were considered and were altruistic in enabling students to learn. They prized professional behaviour and expected a good quality experience of BST with the supervision of qualified professionals. Women reiterated that they should be valued as “Patient as Teacher”.

## Supplementary information

**Additional file 1.** Interview Guide. Interview Guide for Semi-structured interviews of patients participating in Bedside Teaching.

## Data Availability

The datasets generated and/or analysed during the current study are not publicly available due to the qualitative nature of the work but are available from the corresponding author on reasonable request.

## Abbreviations

AMEE: Association for Medical Education in Europe
BST: Bedside Teaching
SRQR: Standards for reporting of Qualitative Research
