# Supplementary material for: Patient perspectives of bedside teaching in an obstetrics, Gynaecology and neonatology hospital
Source: BMC Med Educ. 2020 Apr 15;20:111. doi: 10.1186/s12909-020-02016-5 (PMC7158153; doi:10.1186/s12909-020-02016-5)
Supplement: Supplementary file 1 — Additional file 1. Interview Guide. Interview Guide for Semi-structured interviews of patients participating in Bedside Teaching. [file 12909_2020_2016_MOESM1_ESM.docx]

Title: Interview Guide

Description: Interview Guide for Semi-structured interviews of patients participating in Bedside Teaching

| **Questions**  **Engagement question**  What was your general impression of taking part in the teaching?  **Expectations**  What were your expectations of teaching? How were your expectations met? How could they be better met?  **Consent**  What would affect your decision to participate?  **Impact on the woman**  How useful did you find taking part in this teaching? Did you learn something you didn’t know? Did your understanding of your treatment change from the teaching?  **Respect, privacy, confidentiality**  How did you feel about students taking your history? Did you feel you were included in the discussion? Was your privacy and confidentiality respected?  **The Students**  How did you feel about students performing an examination? Would your willingness to participate in teaching change if the teaching was for doctors (looking to specialise) rather than medical students? Or for midwives?  **Your personal experience**  How can we improve the teaching for you or other patients? Do you feel you have a place in educating student doctors? Midwifery students? Nursing students? Junior Doctors? If you could change anything about the teaching what would it be?  **Exit questions**  Is there anything else you would like to say about your experience about the teaching? (Why you liked/disliked it?) Of all things discussed today, what do you think is most important? Have we missed anything?  **Other talking points**  Probes  Can you talk more about that? / Please tell me more about that Help me understand what you mean by that… Can you give an example of that? Can you tell me something else about that? |
| --- |
